# Lymphatic uptake and pharmacokinetics of lipid conjugated brush PEG polymers is altered by interactions with albumin and lipoproteins

**DOI:** 10.3389/fphys.2025.1610791

**Published:** 2025-06-27

**Authors:** Mohammad Abdallah, Ian K. Styles, Alexander Mörsdorf, James L. Grace, John F. Quinn, Michael R. Whittaker, Natalie L. Trevaskis

**Affiliations:** ^1^ Drug Delivery, Disposition and Dynamics, Monash Institute of Pharmaceutical Sciences, Monash University, Parkville, VIC, Australia; ^2^ Department of Chemical Engineering, Faculty of Engineering, Monash University, Clayton, VIC, Australia; ^3^ Baker Heart and Diabetes Institute, Melbourne, VIC, Australia

**Keywords:** lymphatic transport, lymphatic delivery, lipid, polymer, albumin, lipoprotein, pharmacokinetics

## Abstract

**Introduction:**

Increased recognition of the role of lymphatics in disease has brought increased focus on the design of lymph-directed delivery systems for imaging agents and therapeutics. Previously, we developed novel brush polyethylene glycol (PEG) polymers functionalized with different lipids and investigated their lymphatic uptake, plasma pharmacokinetics and tissue biodistribution. Diacylglycerol-conjugated brush PEG polymers had enhanced lymphatic uptake and extended plasma elimination half-life after both intravenous (IV) and subcutaneous (SC) administration compared with polymers functionalized with single hydrocarbon chain lipids. These differences in *in vivo* trafficking were suggested to occur as a consequence of association with endogenous lipid transport pathways, including albumin and lipoproteins. Herein we investigate the impact of pre-mixing the polymers with albumin or high density lipoproteins (HDLs) on their lymphatic uptake, pharmacokinetics and biodistribution, and detail the mechanisms underpinning the polymers *in vivo* trafficking.

**Methods:**

First, the impact of pre-mixing diacylglycerol-conjugated PEG polymers (2C12-PEG and 2C18-PEG) with defatted rat serum albumin (RSA) or HDL on the polymers’ IV and SC plasma pharmacokinetics, SC lymph uptake and/or biodistribution was investigated. Next, a mechanistic study confirmed the impact of *in situ* association of 2C18-PEG with endogenous HDL particles on polymer biodistribution by inhibiting the scavenger receptor class B type 1 (SRB1) receptor before SC dosing 2C18-PEG.

**Results:**

Pre-mixing 2C12-PEG with RSA (2C12-PEG/RSA) prolonged the elimination half-life of 2C12-PEG following IV and SC dosing. However, SC lymph transport of 2C12-PEG was reduced by 2C12-PEG/RSA. In contrast, the concentration of 2C18-PEG in plasma, lymph nodes and several tissues increased by pre-mixing with HDL (2C18-PEG/HDL). Unexpectedly, the biodistribution of 2C18-PEG into ipsilateral lymph nodes and adipose tissues at 4 h after dosing was increased in mice pre-dosed with the SRB1 inhibitor, likely due to perturbations in the lipoprotein profile.

**Discussion:**

Overall, administration with albumin and altering lipoprotein trafficking pathways modified the biodistribution and lymphatic uptake of the polymers, supporting that they traffic into lymph in association with lipid trafficking pathways. Increasing the association of delivery systems such as lipidated polymers with HDL trafficking pathways could be a viable means to enhance lymphatic uptake of diagnostic and therapeutic agents for lymphatic diseases.

## 1 Introduction

The lymphatic system is now accepted to contribute not only to physiological processes (fluid and lipid transport, immune function, etc.), but also to the pathophysiology of many diseases, including lymphedema, cancer, infectious, inflammatory and cardiometabolic diseases (Oliver et al., 2020; Petrova and Koh, 2020; Reddiar et al., 2024). Increased appreciation of the involvement of lymphatics in disease has further focused efforts to develop new tools to understand lymphatic function, and diagnose and treat lymphatic diseases. This includes the development of systems to deliver imaging agents, vaccines and therapeutics into the lymphatics (Trevaskis et al., 2015; Schudel et al., 2019; Abdallah et al., 2020; Reddiar et al., 2024). The majority of delivery systems developed to date have relied on size-selective entry into lymphatics as the initial lymphatic capillaries in tissues, which possess open button-like junctions between endothelial cells, are far more permeable than blood capillaries (Oliver et al., 2020; Petrova and Koh, 2020; Reddiar et al., 2024). This enables lymphatic transport of macromolecules, proteins, lipoproteins, nanoparticles and smaller agents that associate *in situ* in tissues with endogenous macromolecules such as albumin and lipoproteins (Trevaskis et al., 2015; Schudel et al., 2019; Abdallah et al., 2020; Reddiar et al., 2024).

Endogenous and exogenous albumin and lipoproteins have been exploited in many advanced drug delivery systems to overcome challenges in drug delivery such as short elimination half-life, inadequate accumulation in target tissues, non-specific distribution to off-target tissues, and solubility issues for highly lipophilic drugs (Larsen et al., 2016; Sobot et al., 2017; Abdallah et al., 2020; Busatto et al., 2020; Rahimizadeh et al., 2020). In the case of exogenous albumin, therapeutic or imaging agents can be covalently or non-covalently attached prior to delivery (Larsen et al., 2016; Abdallah et al., 2020). A prominent example is the marketed product Abraxane® where the anti-tumor chemotherapeutic drug paclitaxel is physically encapsulated with albumin leading to an improved biodistribution, efficacy and safety profile (Larsen et al., 2016). As far as we are aware the lymphatic uptake of Abraxane or similar products has not been measured previously but is expected to be relatively high. In recent years, many studies have reported the design of systems that associate *in situ* with endogenous albumin following administration leading to improved pharmacokinetics (e.g., longer elimination half-life) and biodistribution (Abdallah et al., 2020; Zaragoza, 2023). In these systems, albumin binding moieties (ABMs) such as lipids, blue dyes and drugs are covalently attached to therapeutics, vaccines or polymer delivery systems to encourage association with albumin (Larsen et al., 2016; Abdallah et al., 2020). Following interstitial injection, these types of systems can be taken up directly into the lymphatics through hitchhiking on endogenous albumin transport pathways into lymph (Larsen et al., 2016; Abdallah et al., 2020). The type of ABM is important in dictating the extent of lymphatic uptake. For example, different types of lipids have different albumin binding affinities and thus propensities to promote lymphatic uptake. An early study on this topic by Liu *et al* reported that conjugation of vaccine adjuvant cytosine-guanine-oligodeoxynucleotides (CpG-ODNs) to long chain (C18) diacyl lipids led to stronger albumin binding affinity and enhanced lymph node accumulation than conjugation to fatty acid (stearoyl, C18), cholesterol or shorter chain length diacyl lipids (C12, C14, C16) (Liu et al., 2014).

Enhanced lymphatic delivery can also be achieved through association with lipid and lipoprotein trafficking pathways into the lymphatics. For example, oral administration of highly lipophilic drugs or prodrugs can lead to lymphatic uptake through association with chylomicron transport pathways into the intestinal lymphatics (Yáñez et al., 2011; Trevaskis et al., 2015; Han et al., 2021; Elz et al., 2022). In addition to chylomicrons, endogenous low-density lipoproteins (LDL) and high-density lipoproteins (HDL) particles have been exploited to extend the elimination half-life and to promote lymph uptake of pharmaceutical agents following parenteral delivery (de Smidt et al., 1991; Gracia et al., 2022). For example, the association of a cholesterol-conjugated antisense oligonucleotide (OND) with endogenous LDL and HDL particles was considered the mechanism behind the prolonged intravenous (IV) elimination half-life (9–11 min) of cholesterol-conjugated OND compared with unconjugated OND (<1 min) (de Smidt et al., 1991). Our group also compared the lymphatic uptake of cholesterol-conjugated cytosine phosphoguanine (CpG) adjuvant to unconjugated CpG following SC administration (Gracia et al., 2022). The cholesterol-conjugated CpG adjuvant had higher lymph uptake (∼7-fold) compared with the unconjugated CpG adjuvant, which appeared to be a result of the *in situ* association of the cholesterol-conjugated CpG adjuvant with endogenous HDL particles (Gracia et al., 2022).

Recently, we designed a new delivery system to enhance lymphatic uptake of therapeutics, imaging agents or vaccines that consists of a brush polyethylene glycol (PEG) polymer conjugated to lipids that can associate with albumin and lipoproteins (Abdallah et al., 2024a; Abdallah et al., 2024b). We prepared five different brush PEG polymers attached to different lipid components: short-chain or medium-chain hydrocarbon tails (1C2-PEG and 1C12-PEG, respectively), cholesterol (Cho-PEG), or diacylglycerols made of medium-chain or long-chain fatty acids (2C12-PEG or 2C18-PEG, respectively). We found that each polymer had a different association pattern with albumin and the lipoprotein species present in rat plasma and lymph (Abdallah et al., 2024a; Abdallah et al., 2024b). 2C12-PEG generally appeared to have the highest association with albumin, whereas 2C18-PEG appeared to have the highest association with HDL particles (Abdallah et al., 2024b). The diacylglycerol-conjugated brush PEG polymers also displayed enhanced lymphatic uptake, extended plasma elimination half-life, and distinct biodistribution profiles after IV and subcutaneous (SC) administration to rodents (Abdallah et al., 2024a; Abdallah et al., 2024b). These appealing features of the polymers are mediated by their hitchhiking on natural lipid carriers, which is facilitated by conjugating the polymers with lipids. The lipidated brush PEG polymers thus represent promising new tools to deliver attached imaging agents, therapeutics or vaccines to lymph. Apart from lipidation, the stealth effect of these polymers would render them excellent drug delivery platforms to improve the pharmacokinetics of pharmaceutical agents (especially agents with short plasma half-lives) (Wen et al., 2023).

The aims of the current study are two-fold. First, we evaluate the impact of pre-mixing the polymers with exogenous albumin or HDL on the IV and SC plasma pharmacokinetics, lymph uptake and biodistribution. We chose 2C12-PEG and 2C18-PEG for the studies since the elimination half-life and lymph uptake of 2C12-PEG and 2C18-PEG were higher than 1C2-PEG and 1C12-PEG. We hypothesized that pre-mixing with albumin or HDL may further improve the *in vivo* trafficking of the polymers (i.e., prolong the elimination half-life and enhance lymphatic uptake). Differences in the lymphatic uptake and pharmacokinetics on pre-mixing with albumin and HDL may also provide mechanistic insight into the main trafficking pathway that promotes lymph uptake of the polymers. This leads to our second aim which is to reveal more about the mechanisms driving the *in vivo* transport of the polymers, and particularly their lymphatic uptake. As well as pre-mixing the polymers with albumin or HDL, we evaluate the lymph node uptake and biodistribution of 2C18-PEG in mice pre-dosed with block lipid transport-1 (BLT-1), an inhibitor of scavenger receptor class B type 1 (SRB1) which mediates the entry of HDL into lymphatics and the transfer of lipids between HDLs and cells (Lim et al., 2013). Overall, the studies strongly suggest that hitchhiking of the polymers on lipid trafficking pathways leads to lymphatic uptake. The distinct tissue biodistribution profile of the polymers is likely to be useful for developing lymph-specific imaging agents and therapeutics to treat lymphatic diseases.

## 2 Materials and methods

### 2.1 Materials

Dulbecco’s phosphate buffered saline (DPBS) 1× (pH 7.4), defatted rat serum albumin (RSA), BLT-1, glycerol standard solution (0.26 mg/mL), corn oil, sterile Corning® syringe filters (pore size 0.2 μm), Costar® 24-well transparent plates (flat clear bottom) and Corning® 96-well black microplates (flat clear bottom) were purchased from Sigma-Aldrich (MO, United States). Amplex™ red cholesterol assay kit was purchased from Invitrogen (Carlsbad, CA, United States). Ethanol absolute (analytical reagent) and Infinity™ triglycerides reagent kit were purchased from Thermo Fisher Scientific Australia (VIC, Australia). Amicon® Ultra-0.5 Centrifugal Filter 30K Devices were purchased from Merck Millipore Ltd. (Darmstadt, Germany). Vivaspin® 2 Centrifugal Concentrator Polyethersulfone (molecular weight cut-off (MWCO) 10 kDa) was purchased from Sartorius AG (Göttingen, Germany). Heparin (10 0 0 IU/mL) and carprofen (50 mg/mL) were purchased from Clifford Hallam Healthcare Pty. Ltd. (VIC, Australia). Isoflurane solution for general anesthesia and Lethabarb® (325 mg/mL pentobarbitone sodium) were purchased from Pro-Vet (VIC, Australia). Chlorhex-C® (5% w/v chlorhexidine gluconate) antiseptic/disinfectant concentrate was purchased from Jurox Animal Health (NSW, Australia). Standard rodent diet (7% w/w fat) (AIN93G) was purchased from Specialty Feeds (Western Australia, Australia).

### 2.2 Structure, synthesis and characterization of the polymers

**FIGURE 1:**
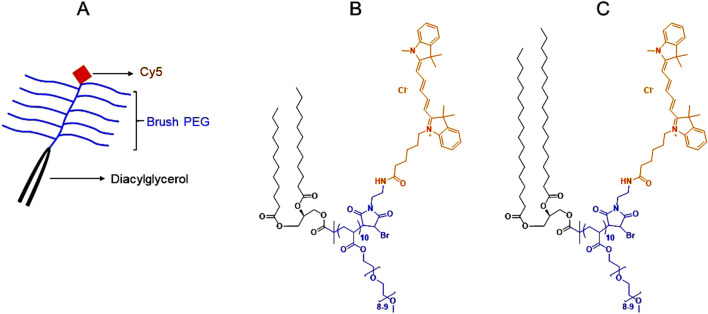
Color-coded overall and chemical structures of diacylglycerol-conjugated brush PEG polymers (molecular weight: ∼6 kDa). **(A)**: A schematic showing the overall structure of the diacylglycerol-conjugated brush PEG polymers labeled with Cyanine 5 (Cy5). **(B)**: The chemical structure of Cy5-labeled medium-chain (2C12) diacylglycerol-conjugated brush PEG. **(C)**: The chemical structure of Cy5-labeled long-chain (2C18) diacylglycerol-conjugated brush PEG. Black represents the diacylglycerol moiety, blue represents the brush PEG backbone and orange represents Cy5. Full description of the synthesis and characterization of the polymers was provided in (Abdallah et al., 2024b).

The synthesis and characterization of Cyanine 5 (Cy5) labelled brush PEG polymers (molecular weight: ∼6 kDa) conjugated with medium-chain (2C12) or long-chain (2C18) diacylglycerols (Figure 1) were described previously (Abdallah et al., 2024b). Cy5 was coupled with the polymers to enable their quantification in biological fluids and tissues.

### 2.3 Polymer selection and preparation for *in vivo* dosing

2C12-PEG and 2C18-PEG were selected for our studies as our previous studies (Abdallah et al., 2024a; Abdallah et al., 2024b) demonstrated that these polymers display higher albumin and HDL association, lymph uptake and plasma half-life compared with brush PEG polymers conjugated with single short-chain (1C2) or medium-chain (1C12) hydrocarbon tails. While Cho-PEG had a good association with albumin and lipoproteins, its lymph uptake was surprisingly lower than 2C12-PEG and 2C18-PEG (Abdallah et al., 2024a). Thus Cho-PEG was not used in our present study.

To prepare 2C12-PEG/RSA, 2C12-PEG (10 mg/mL) was mixed with a defatted RSA solution (50 mg/mL in DPBS). In SC pharmacokinetic and lymph uptake studies, 150 μL of the mixture was administered SC into the right hind leg of rats. In IV pharmacokinetic studies, 150 μL of the mixture was diluted with 350 μL of DPBS, then the 500 μL was administered IV over 30 s through the jugular vein cannula. In the biodistribution studies in mice, 50 μL containing 2C12-PEG (2 mg/mL) and defatted RSA (10 mg/mL) was administered SC in the right hind leg of mice. The dose of 2C12-PEG in all studies was ∼5 mg/kg with a 2C12-PEG:RSA molar ratio of ∼2.1:1 in all formulations.

To prepare 2C18-PEG/HDL, the L-HDL fraction (density 1.06 - 1.10 g/mL) was first separated from rat plasma as described previously (Gracia et al., 2020). Next, L-HDL particles were isolated by ultrafiltration using Amicon® Ultra-0.5 Centrifugal Filter 30K Device (Merck Millipore Ltd., Darmstadt, Germany) with centrifugation force of 14,000 xg at 37°C for 10 min. After that, the L-HDL particles were washed thrice with DPBS using the same centrifugation settings as in the ultrafiltration method. L-HDL particles were then diluted with DPBS to make a 10 mg/mL solution, which was filtered with a sterile Corning® syringe filter (pore size 0.2 μm) made of regenerated cellulose membrane (Sigma-Aldrich, MO, United States). The filtered L-HDL solution (10 mg/mL) was used as a diluent for 2C18-PEG (2 mg/mL), which was dosed (50 μL) SC to mice.

After mixing 2C12-PEG and 2C18-PEG with RSA and L-HDL particles, the unattached fraction of the polymers was not filtered. We were not able to do this step as we found that the polymers adsorbed substantially to various types of filter membrane during ultrafiltration. These filter membranes were made of either regenerated cellulose (Amicon® Ultra-0.5 Centrifugal Filter 30K Device (Merck Millipore Ltd., Darmstadt, Germany)) or polyethersulfone (Vivaspin® 2 Centrifugal Concentrator Polyethersulfone (MWCO 10 kDa) (Sartorius AG, Göttingen, Germany)).

### 2.4 Animal studies

All animal studies were approved by the Monash Institute of Pharmaceutical Sciences (MIPS) animal ethics committee. Rats and mice were obtained from the Monash Animal Research Platform (Victoria, Australia) and were acclimatized for at least 7 days before *in vivo* studies. During acclimatization, rats and mice were housed in standard husbandry conditions with free access to standard fat diet (7% w/w fat) and water under a 12-h light-dark cycle. IV and SC pharmacokinetic studies and lymph uptake studies for 2C12-PEG/RSA were conducted in male Sprague Dawley rats (age: 8–12 weeks, weight: ∼250–350 g), while biodistribution studies were conducted in male C57BL/6 mice (age: 7–10 weeks, weight: ∼20–25 g), as described previously (Abdallah et al., 2024a; Abdallah et al., 2024b). The results of the rat studies were compared with the results of matching studies for 2C12-PEG published previously (Abdallah et al., 2024a; Abdallah et al., 2024b). Rats were used in plasma pharmacokinetics and lymph uptake studies as lymph cannulation is very challenging in mice and the volume of thoracic lymph that can be collected from mice is very small (Ionac, 2003; Caliph et al., 2015). On the other hand, because of the small size of mice their organs can be better imaged with *in vivo* imaging systems (IVIS) (PerkinElmer, Inc., Waltham, MA, United States) compared to rats, enabling determination of tissue biodistribution (Chen and Thorne, 2012).

Noteworthy, randomization of animals and blinding in our study were not applied as we used healthy control animals, of the same gender, and had similar age and weight. Therefore, the absence of randomization and blinding did not weaken the study design and would unlikely affect the validity of our findings. Also, it is important to highlight that, in accordance with the literature and because of the low variability among animals, a sample size of 3 was used in lymph uptake and plasma pharmacokinetic studies in rats, while a sample size of 4 was used in biodistribution studies in mice (Schipper et al., 2007; Willmann et al., 2008; England et al., 2017; Wooten et al., 2017; Kumar et al., 2018; Klapproth et al., 2020; Fu et al., 2021; Tusé et al., 2022; Singh et al., 2023; Muehlberg et al., 2024; Wang et al., 2024). Importantly, the 3R principle (Replacement, Reduction, and Refinement) in animal research is followed strictly in our institution to reduce the number of animals in research (Lee et al., 2020). An additional mouse was included in biodistribution studies in mice as the data in biodistribution studies was represented as fluorescence intensity. Fluorescence intensity can be variable among groups, compared with plasma and lymph concentrations, mandating the inclusion of an additional animal in each group to make the comparison between the groups feasible.

### 2.5 Experimental design of pharmacokinetic and lymph uptake studies after IV and/or SC dosing in rats

Rat SC and IV plasma pharmacokinetic studies and lymph uptake studies (following SC dosing) were performed to determine if the trafficking of 2C12-PEG is altered by pre-mixing with exogenous defatted RSA (2C12-PEG/RSA). In these, the right carotid artery of rats was cannulated to facilitate intermittent blood sampling. In IV plasma pharmacokinetic studies and lymph uptake studies, the left jugular vein was also cannulated to enable delivery of the IV formulations (2C12-PEG and 2C12-PEG/RSA) in IV plasma pharmacokinetic studies, or normal saline in lymph uptake studies to facilitate hydration of rats. In lymph uptake studies, the thoracic duct was cannulated to enable the continuous collection of lymph fluid. Briefly, cannulation procedures were conducted on the first day under anesthesia, which was induced with isoflurane 5% v/v in medical oxygen or Carbogen then maintained with isoflurane 1.5%–2.5% v/v in medical oxygen or Carbogen. All skin incision sites were shaved using a trimmer then cleaned with an antiseptic solution containing 0.5% chlorhexidine and 70% ethanol. Before making skin incisions, 50 μL of bupivacaine 0.5% and 5 mg/kg carprofen were administered SC at the incision sites and in the hind flank, respectively, to minimize post-operative pain. After surgeries, isoflurane anesthesia was disconnected and rats were placed in a BASi Raturn® cages, in which they were allowed to regain consciousness and to recover overnight. Water was provided to rats after surgeries while they were fasted from food until 4 h after dosing with 2C12-PEG formulations, which was performed on the day following surgeries. In the lymph uptake studies, 5 mg/kg carprofen was administered in the hind flank of lymph cannulated rats twice daily until the last day of the lymph uptake. Post-operative carprofen analgesia was not given to lymph intact rats as blood cannulation surgeries are less invasive compared to lymph cannulation.

In SC plasma pharmacokinetic studies and lymph uptake studies, rats were anesthetized briefly with isoflurane (5% v/v in medical oxygen or Carbogen for induction and 1.5%–2.5% v/v in medical oxygen or Carbogen for maintenance). After that, the right hind leg of rats was cleaned with an antiseptic solution (composed of 0.5% chlorhexidine and 70% ethanol). Then rats were dosed SC with 2C12-PEG formulations, after which anesthesia was cut and rats were returned to their cages. On the other hand, in IV plasma pharmacokinetic studies, rats were dosed while they were conscious through the jugular vein cannula.

In IV pharmacokinetic studies, blood samples (∼250 μL each) were collected from rats at the following timepoints: pre-dose, 1, 5, 15 and 30 min plus 1, 2, 4, 8, 24, 30 and 48 h after dosing. In SC pharmacokinetic studies, blood samples (∼250 μL each) were collected from rats at the following timepoints: pre-dose, 30 min and 1, 2, 3, 4, 6, 8, 24, 32, 46 and 48 h after dosing. Blood samples (∼ 250 μL each) were collected in lymph uptake studies from rats at the following timepoints: pre-dose, 30 min and 1, 2, 3, 4, 6, 8, 24, 30, 32 h after SC dosing. Further, in lymph uptake studies, lymph was collected continuously after cannulation of the thoracic duct until SC dosing, and also at the following time intervals after SC dosing: 0–30 and 30–60 min then 1–1.5, 1.5–2, 2–2.5, 2.5–3, 3–3.5, 3.5–4, 4–6, 6–8, 8–24 and 24–32 h. Blood samples were collected in 1.5 mL Eppendorf tubes pre-filled with 5–10 μL of heparin (1000 IU/mL). On the other hand, the volume of heparin (1000 IU/mL) in lymph collected tubes was determined based on the expected volume of collected lymph (typical rate 1–2 mL/h). Prior to lymph collection, 10 μL of heparin (1000 IU/mL) was added in lymph collecting tubes for each 1 mL of lymph.

At the end of animal studies, ∼10 mL of blood was collected from rats through the carotid artery cannula and placed in a 15 mL centrifuge tube pre-filled with 100 μL of heparin (1000 IU/mL). After terminal blood collection, rats were humanly killed with 100 mg/kg pentobarbitone sodium administered into the cannulated carotid artery or jugular vein. Terminal blood collection and humane killing were performed under isoflurane anesthesia (5% v/v in medical oxygen or Carbogen for induction and 1.5%–2.5% v/v in medical oxygen or Carbogen for maintenance). After euthanasia, popliteal, inguinal and iliac lymph nodes from the ipsilateral (dosing) and contralateral (non-dosing) sides were collected.

### 2.6 Experimental design of biodistribution studies in mice

To investigate the impact of co-administration with HDL particles or blocking HDL receptors on the lymph node uptake and biodistribution of 2C18-PEG, biodistribution studies were conducted in two groups of male C57BL/6 mice (age: 7–10 weeks, weight: ∼20–25 g). In the first group, 2C18-PEG was pre-mixed with L-HDL particles and delivered SC to mice. Then mice were killed at 4 or 24 h after dosing then lymph nodes, several tissues and major organs were collected. In the second group of studies, male C57BL/6 mice (age: 7–10 weeks, weight: ∼20–25 g) were pre-dosed orally with BLT-1, an inhibitor of SRB1 which facilitates the entry of HDL into lymphatic vessels and transfer of lipids between HDL particles and cells. A dose of 1 mg/kg of BLT-1 was delivered by oral gavage (200 μL each dose) in accordance with the literature (Lino et al., 2015). A vehicle consisting of DMSO-corn oil (10:90, v/v) was used to dissolve BLT-1 as it is poorly soluble in water. After 30 min of dosing mice orally with BLT-1, mice were dosed SC with 2C18-PEG, then humanely killed after 4 h. After that, lymph nodes, several tissues and major organs were collected. The results of these biodistribution studies were compared with the results of matching studies for 2C18-PEG published previously (Abdallah et al., 2024a). At the end of the mouse studies, cardiac puncture to collect a terminal blood sample (∼1 mL) which was placed in a 1.5 mL Eppendorf tubes pre-filled with 5–10 μL of heparin (1000 IU/mL). Mice were then killed by cervical dislocation. Both blood collection and humane killing were performed under isoflurane anesthesia (5% v/v in medical oxygen or Carbogen for induction and 1.5%–2.5% v/v in medical oxygen or Carbogen for maintenance). Popliteal, inguinal and iliac lymph nodes from the ipsilateral (dosing) and contralateral (non-dosing) sides were then collected from the mice. In addition, several tissues and organs were collected including skin, SC adipose tissue and skeletal muscle from the ipsilateral and contralateral sides, kidneys, liver, heart, spleen, lungs and brain. SC adipose tissue from the ipsilateral side of mice was collected at the 24-h timepoint only.

### 2.7 Biological fluid and tissue sample processing and analysis

Following collection, mouse and rat blood and rat lymph were centrifuged (5,890 xg for 5 min at room temperature) to separate plasma and lymph fluid without cells. Furthermore, collected lymph nodes from rats were homogenized, as described previously (Abdallah et al., 2024a), using a MP bio FasPrep 24- G system (MP Biomedicals, Seven Hills, NSW, Australia). Concisely, each lymph node was homogenized in a screw cap 0.5 mL microtube pre-filled with four ceramic beads (1.4 mm), 50–100 mg of garnet beads (0.15 mm) and 100–200 μL of lysis buffer. The lymph node homogenate was then centrifuged (4,500 xg for 5 min at room temperature). All samples of separated plasma, centrifuged lymph and homogenized lymph nodes were stored at −20°C until analysis.

A fluorescence assay was used to quantify the Cy5 labelled polymers in 100 μL samples of biological fluids and homogenized lymph nodes (mixed 1:3 with lysis buffer) (Abdallah et al., 2024a; Abdallah et al., 2024b). The samples (100 μL each) were placed in Corning® 96-well black microplates (flat clear bottom), then the fluorescence from the top of the plate was measured by a PerkinElmer EnSight™ multimode plate reader using 642 and 665 nm as excitation and emission wavelengths, respectively. The fluorescence assay was validated for plasma and lymph samples using two replicates of six standard solutions (0.25, 0.5, 1, 2.5, 5, 10 μg/mL) and six replicates of four quality control samples (0.25, 1, 4, 8 μg/mL) diluted in blank plasma and lymph, respectively. Whereas the assay was validated for homogenized lymph nodes (mixed 1:3 with lysis buffer) using two replicates of four standard solutions (0.25, 0.5, 1, 5 μg/mL) and six replicates of three quality control samples (0.25, 1, 4 μg/mL) of the polymers diluted in homogenized blank rat lymph nodes (mixed with 1:3 lysis buffer). Accuracy and precision of the assay were considered acceptable as they were within ±10% for high concentrations and ±15% for the lowest concentration.

Regarding collected intact lymph nodes, tissues and organs from mice, they were placed in 24-well Costar® 24-well microplates (clear tissue culture-treated) and their radiant efficiency was measured with *in vivo* imaging systems (IVIS) (PerkinElmer, Inc., Waltham, MA, United Sr) at 640 nm and Cy5.5 as excitation and emission filters, respectively. Background radiant efficiency of lymph nodes, tissues and organs was measured for mice injected with DPBS. This background signal was subtracted from the radiant efficiency of matching lymph nodes, tissues and organs collected from mice injected with 2C12-PEG and 2C18-PEG formulations. A detailed description of the methods and settings were provided previously (Abdallah et al., 2024a; Abdallah et al., 2024b).

### 2.8 Analysis of plasma pharmacokinetic and lymph uptake data

Plasma pharmacokinetic and lymph uptake parameters were calculated using standard non-compartmental analysis as described previously (Abdallah et al., 2024a; Abdallah et al., 2024b). Equations used to calculate non-compartmental plasma pharmacokinetic parameters after IV and SC dosing are summarized in Supplementary Table S1.

The mass of each lymph sample was measured and was used to calculate the volume of lymph samples by assuming a lymph density of 1 g/mL. The lymph flow rate for each lymph collection interval was calculated by dividing the volume of collected lymph by the time interval of collection. The multiplication of the normalized concentration of 2C12-PEG in each lymph sample by the volume of collected lymph was used to calculate the mass transport of 2C12-PEG into lymph during each time interval. Moreover, the percentage of 2C12-PEG dose transported into lymph was determined by dividing the mass transport in lymph by the dose administered and multiplying by 100%. Further, lymph to plasma concentration ratios were calculated by dividing the concentration of 2C12-PEG in lymph collected over the determined time intervals by 2C12-PEG concentration in plasma from lymph intact rats at the same time interval. Additionally, the percent of the 2C12-PEG retained in the lymph nodes at the end of lymph uptake studies (at 32 h after dosing) was calculated as follows:

$$\text{Percent}\;\text{retained}\;\text{in}\;\text{the}\;\text{lymph}\;\text{nodes}=\frac{\text{Mass}\;\text{in}\;\text{lymph}\;\text{nodes}}{\text{Mass}\;\text{in}\;\text{lymph}\;\text{node}+\text{Mass}\;\text{in}\;\text{lymph}\;\text{fluid}}\times 100\%$$

The concentrations of 2C12-PEG in plasma and lymph were dose normalized as per the following equation:

$$\text{Normalised}\;\text{plasma}\;\text{or}\;\text{lymph}\;\text{concentration}=\frac{\text{expected}\;\text{dose}}{\text{measured}\;\text{dose}}\times \text{measured}\;\text{plasma}\;\text{or}\;\text{lymph}\;\text{concentration}$$

### 2.9 Determination of triglyceride and cholesterol levels in mouse plasma

The concentrations of triglyceride and total cholesterol in mouse plasma were determined by enzyme-coupled reactions using Infinity™ triglycerides reagent kit (TR0100, Thermo Fisher Scientific Australia, VIC, Australia) and Amplex™ red cholesterol assay kit (A12216, Invitrogen, Carlsbad, CA, United States), respectively, following the manufacturers’ instructions. For the triglycerides kit, standard solutions were prepared using a 0.26 mg/mL glycerol standard solution (G7793, Sigma-Aldrich, MO, United States), which is equivalent to 2.5 mg/mL triolein concentration. Whereas for the cholesterol assay kit, a series of standard solutions were prepared using a cholesterol reference standard (2 mg/mL) provided with the kit.

### 2.10 Statistical methods

All data are presented as mean ± SD. An unpaired t-test or two-way analysis of variance (ANOVA) was conducted to compare two groups with one or two independent variables, respectively. To compare three groups, one-way ANOVA was conducted. One-way and two-way ANOVA tests were followed by Tukey’s *post hoc* test to compare between specific groups. The level of statistical significance was set at p ≤ 0.05. Statistical analyses were conducted using GraphPad Prism 9.1.0 software (GraphPad Software Inc., CA, United States).

## 3 Results

### 3.1 Pre-mixing 2C12-PEG with RSA extends its elimination half-life after SC and IV administration

**FIGURE 2:**
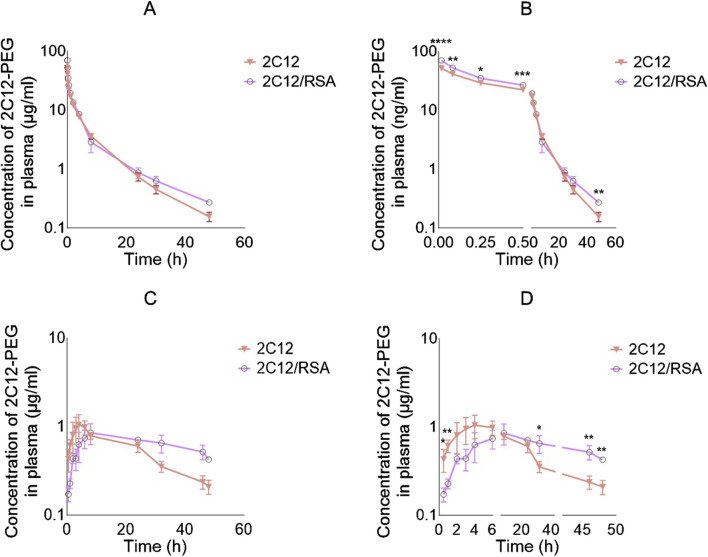
Log plasma concentration versus time curves of 2C12-PEG after IV and SC administration to rats with and without defatted RSA (2C12-PEG/RSA and 2C12-PEG, respectively). **(A)**: IV plasma pharmacokinetic profiles with undivided X-axis. **(B)**: IV plasma pharmacokinetic profiles with X-axis divided into two segments and showing significant differences between the two groups at specific timepoints with asterisks. **(C)**: SC plasma pharmacokinetic profiles with undivided X-axis. **(D)**: SC plasma pharmacokinetic profiles with X-axis divided into three segments and showing significant differences between the two groups at specific timepoints with asterisks. Data are presented as mean ± SD (n = 3 biological replicates per group). Data analysis was performed by unpaired t-test *p ≤ 0.05, **p ≤ 0.01, ***p ≤ 0.001, ****p ≤ 0.0001.

Pre-mixing 2C12-PEG with defatted RSA (2C12-PEG/RSA) had a significant impact on the IV pharmacokinetic profile and the elimination half-life of 2C12-PEG (Figure 2; Table 1). The plasma concentrations of 2C12-PEG after administration of 2C12-PEG/RSA were significantly higher in the first four timepoints (i.e. 1, 5, 15 and 30 min) and the last timepoint (i.e. 48 h) compared to the conventional 2C12-PEG formulation. Furthermore, the elimination half-life of 2C12-PEG was extended significantly from 10.9 to 14.2 h with the use of 2C12-PEG/RSA (Table 1).

**TABLE 1:** Summary of plasma pharmacokinetic and lymph uptake parameters for 2C12-PEG administered IV or SC with or without pre-mixing with RSA.

IV pharmacokinetic parameters	2C12-PEG	2C12-PEG/RSA
AUC_Total_ (μg.h/ml)	130.0 ± 7.9	141.7 ± 11.8
Total clearance (mL/h)	11.6 ± 0.7	10.6 ± 0.9
Vd during the terminal phase (mL)	181.7 ± 11.8	219.3 ± 34.6
Elimination half-life (h)	10.9 ± 0.4^a^	14.2 ± 1.4^a^

SC pharmacokinetic parameters	2C12-PEG	2C12-PEG/RSA
Bioavailability (%)	24.2 ± 3.6^a^	38.0 ± 4.9^a^
Terminal half-life (apparent elimination half-life) (h)	17.0 ± 1.3^a^	36.5 ± 2.4^a^
AUC_Total_ (μg.h/mL)	31.4 ± 4.6^a^	53.8 ± 7.0^a^
Tmax (h)	5.3 ± 1.2	6.7 ± 2.3
Cmax (μg/mL)	1.1 ± 0.3	0.9 ± 0.2

Lymph uptake parameters	2C12-PEG	2C12-PEG/RSA
Percentage of dose transported in lymph	5.9 ± 1.1^a^	3.0 ± 1.2^a^
Percentage of bioavailable dose that was lymphatically transported	24.3 ± 4.5^a^	8.0 ± 3.0^a^
Mass recovered in lymph nodes (µg) 32 h after dosing	4.3 ± 1.6	3.8 ± 1.4
Percentage of retained mass in lymph nodes from total mass transported in lymph^b^	4.6 ± 1.2	7.8 ± 2.0

^a^ significantly different parameter compared with the other group (p ≤ 0.05). AUC_Total_: area under the plasma-concentration time curve from time zero to infinity, Vd, volume of distribution.

^b^ Harvested lymph nodes were the popliteal, inguinal and iliac lymph nodes from the ipsilateral (dosing) and contralateral (non-dosing) sides.

Data are presented as mean ± SD (n = 3 biological replicates). Data analysis was performed by unpaired t-test.

The SC plasma pharmacokinetic profiles and parameters for 2C12-PEG were also substantially altered when pre-mixed with RSA (i.e., 2C12-PEG/RSA) (Figure 2; Table 1). The plasma concentration of 2C12-PEG was higher after dosing the rats with conventional 2C12-PEG formulation compared with 2C12-PEG/RSA at the 0.5 and 1 h timepoints, while the concentration of 2C12-PEG was statistically higher using 2C12-PEG/RSA in the last three timepoints (i.e. 32, 46 and 48 h). However, Cmax and Tmax were not statistically different between the 2C12-PEG/RSA and 2C12-PEG groups.

Compared with conventional 2C12-PEG, the SC administration of 2C12-PEG/RSA prolonged the terminal elimination half-life of 2C12-PEG from 17.0 to 36.5 h, increased total area under the plasma-concentration time curve (AUC_Total_) from 31.4 to 53.8 μg h/ml, and enhanced the bioavailability from 24.2% to 38.0% of dose. Similar to the conventional formulation of 2C12-PEG, 2C12-PEG/RSA appeared to display a flip-flop pharmacokinetic behavior following SC administration (Supplementary Figure S1).

### 3.2 Pre-mixing 2C12-PEG with RSA reduces the lymph uptake of 2C12-PEG

**FIGURE 3:**
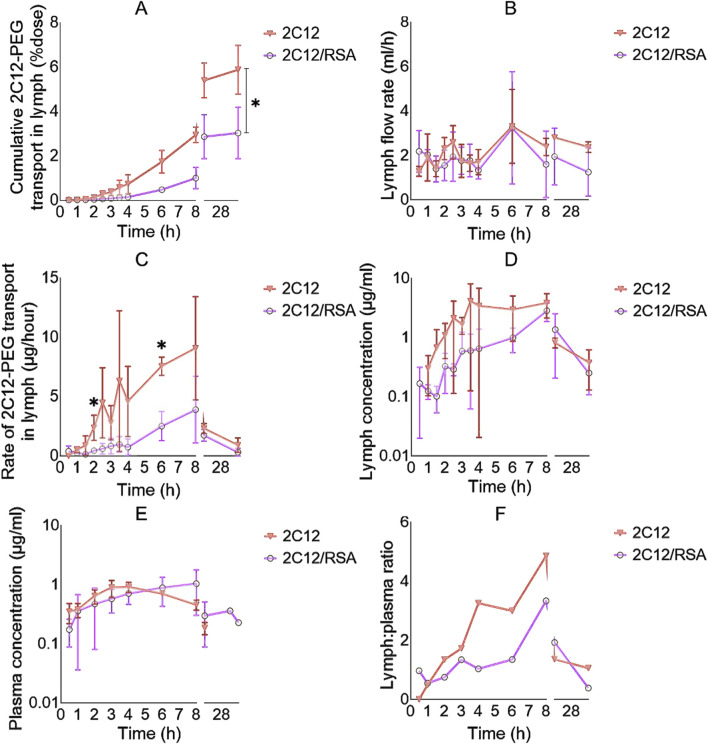
The lymph uptake parameters of 2C12-PEG after IV and SC administration to rats with and without defatted RSA (2C12-PEG/RSA and 2C12-PEG, respectively). **(A)**: The cumulative transport of 2C12-PEG into lymph (% dose) over time. **(B)**: Lymph flow rate (mL/h) over time during the lymph collection intervals. **(C)**: Rate of 2C12-PEG transport into lymph (μg/h) over time. **(D)**: Concentration of 2C12-PEG in lymph (ng/mL) over time. **(E)**: Concentration of 2C12-PEG in plasma (μg/mL) over time. **(F)**: Lymph:plasma concentration ratio of 2C12-PEG over time. Data are presented as mean ± SD (n = 3 biological replicates per group) except for F where data are presented as mean. Data analysis was performed by unpaired t-test with *p ≤ 0.05.

Unexpectedly, the lymph uptake of 2C12-PEG was noticeably reduced when administered following pre-mixing with RSA (i.e., 2C12-PEG/RSA) compared with conventional 2C12-PEG (Figure 3A; Table 1). 2C12-PEG/RSA, however, did not alter the retained mass of 2C12-PEG in lymph nodes at 32 h after dosing, perhaps because this was a late timepoint. The lymph flow (mL/h) for the two groups of rats was in accordance with the literature (Figure 3B) (Ionac, 2003). However, the lymph cannula was completely blocked for one rat in the 2C12-PEG/RSA group after the 24-h timepoint. The rat was then humanely killed and lymph and plasma samples after the 24-h timepoint were not collected. The rate of 2C12-PEG transport (μg/h) into lymph (Figure 3C) was higher with the use of 2C12-PEG alone compared with 2C12-PEG/RSA at the 2 and 6 h timepoints, which resulted in higher cumulative transport of 2C12-PEG into lymph following administration of 2C12-PEG compared to 2C12-PEG/RSA (at the 32-h timepoint). In general, the plasma and lymph concentrations (Figures 3D,E, respectively) of 2C12-PEG following the administration of 2C12-PEG/RSA and 2C12-PEG were not statistically different due to some variability. The lymph:plasma ratio was, however, higher after dosing with 2C12-PEG alone compared with 2C12-PEG/RSA at some timepoints (Figure 3F) suggesting more direct entry into lymph compared to blood for this group.

### 3.3 The biodistribution of 2C12-PEG is slightly affected when it is pre-mixed with rat serum albumin

**FIGURE 4:**
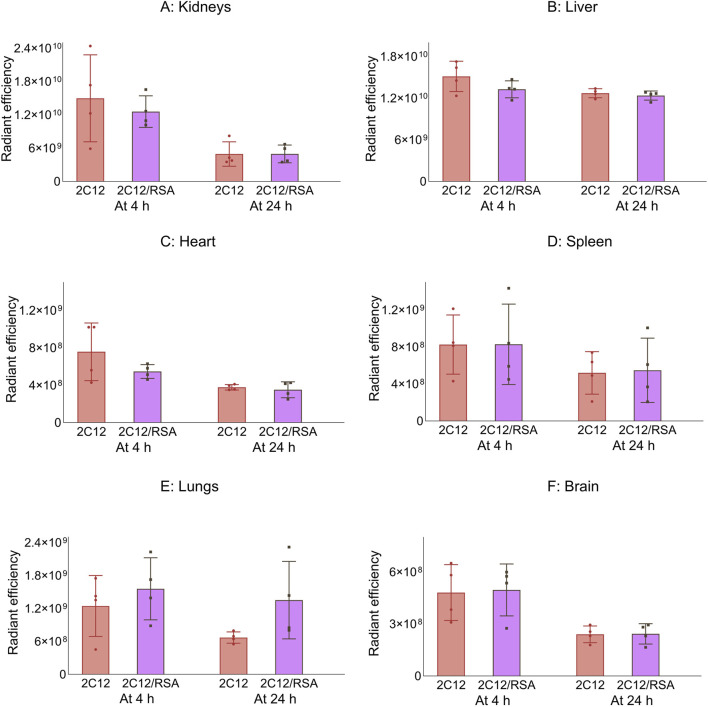
The radiant efficiency of harvested organs and tissues at 4 or 24 h following SC dosing of 2C12-PEG (2C12) or 2C12-PEG/RSA (2C12/RSA) in mice, including **(A)** kidneys, **(B)** liver, **(C)** heart, **(D)** spleen, **(E)** lungs and **(F)** brain. There was no significant difference in the apparent biodistribution pattern into major organs between the two 2C12-PEG formulations. Data are presented as mean ± SD (n = 4 biological replicates per group). Data analysis was performed by unpaired t-test.

**FIGURE 5:**
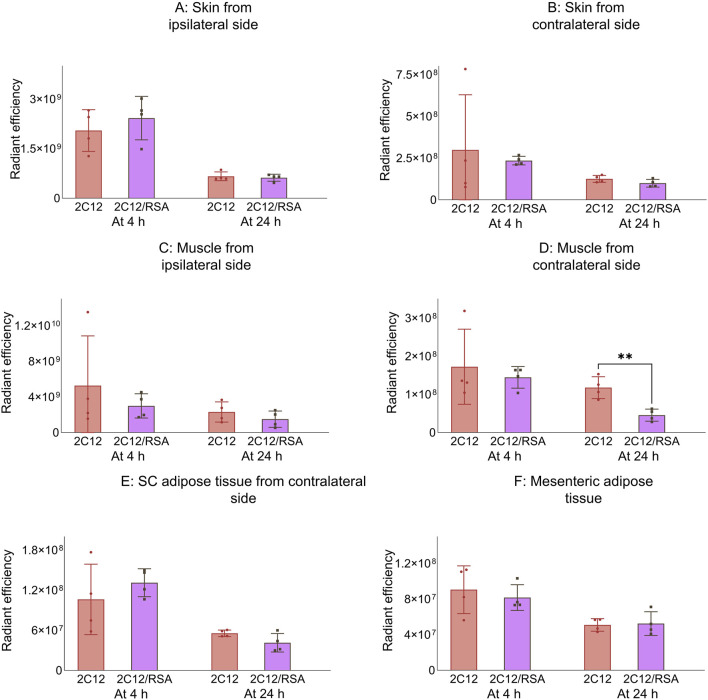
The radiant efficiency of harvested tissues at 4 or 24 h following SC dosing of 2C12-PEG (2C12) or 2C12-PEG/RSA (2C12/RSA) in mice, including **(A)** skin from the dosing site, **(B)** skin from the contralateral side, **(C)** muscle from the dosing site, **(D)** skeletal muscle from the contralateral side, **(E)** SC adipose tissue from the contralateral side and **(F)** mesenteric adipose tissue. Data are presented as mean ± SD (n = 4 biological replicates per group). Data analysis was performed by unpaired t-test with **p ≤ 0.01.

**FIGURE 6:**
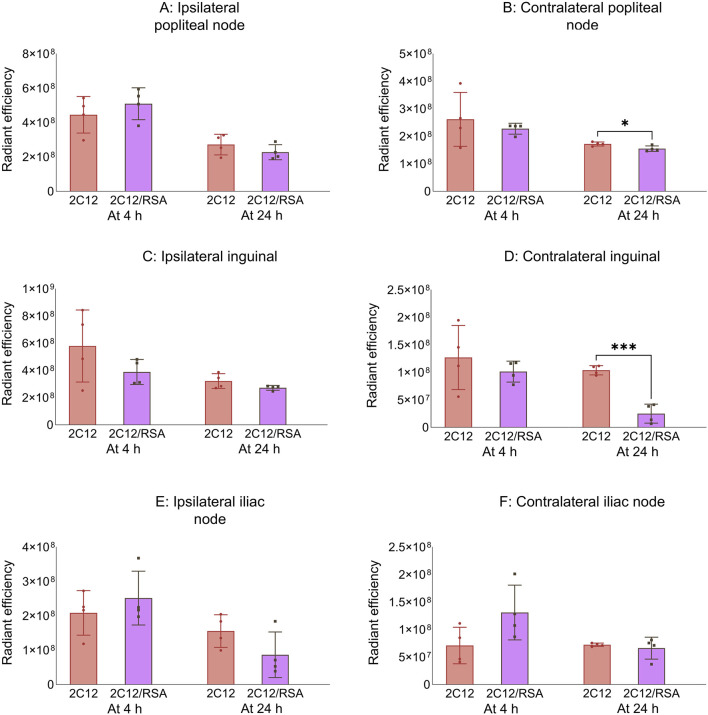
The radiant efficiency of harvested tissues at 4 or 24 h following SC dosing of 2C12-PEG (2C12) or 2C12-PEG/RSA (2C12/RSA) in mice, including **(A)** popliteal lymph nodes from the ipsilateral side, **(B)** popliteal lymph nodes from the contralateral side, **(C)** inguinal lymph nodes from the ipsilateral side, **(D)** inguinal lymph nodes from the contralateral side, **(E)** iliac lymph nodes from the ipsilateral side and **(F)** iliac lymph nodes from the contralateral side. Data are presented as mean ± SD (n = 4 biological replicates per group). Data analysis was performed by unpaired t-test with *p ≤ 0.05, ***p ≤ 0.001.

Next, the biodistribution of 2C12-PEG was compared in mice at 4 and 24 h after SC dosing with or without RSA. The biodistribution was slightly changed on dosing with RSA (Figures 4–6). Popliteal lymph nodes, inguinal lymph nodes and skeletal muscles from the contralateral side of mice injected with 2C12-PEG/RSA had statistically less radiant efficiency compared with the same nodes and muscles harvested from mice injected with 2C12-PEG at the 24-h timepoint. This is consistent with the lower lymphatic uptake observed in lymph cannulated rats (Figure 3A). Importantly, the plasma concentration of 2C12-PEG was statistically higher in the 2C12-PEG/RSA group compared with the 2C12-PEG group at 24 h after dosing, also consistent with the data in rats (Table 2). There was, however, no significant difference in the radiant efficiency of the adipose tissue near the injection site at 24 h following dosing between the two groups (Supplementary Figure S2).

**TABLE 2:** Plasma concentrations of 2C12-PEG at the end of biodistribution studies of the two 2C12-PEG formulations (2C12-PEG and 2C12-PEG/RSA).

Plasma concentration (μg/mL)	Formulation	
	2C12-PEG	2C12-PEG/RSA
4 h timepoint	0.9 ± 0.4	1.3 ± 0.1
24 h timepoint	0.4 ± 0.09^a^	0.6 ± 0.04^a^

^a^ significantly different compared with the other group (P ≤ 0.05).

Data are presented as mean ± SD (n = 4 biological replicates). Data analysis was performed by unpaired t-test.

### 3.4 The biodistribution of 2C18-PEG is significantly affected when it is pre-mixed with HDL

**FIGURE 7:**
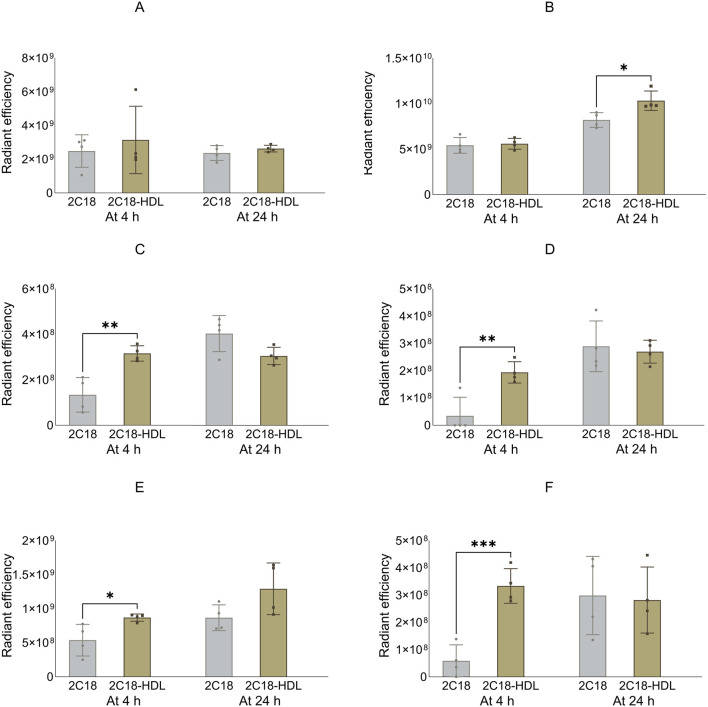
The radiant efficiency of harvested organs at 4 or 24 h following SC dosing of 2C18-PEG (2C18) or 2C18-PEG/HDL (2C12/HDL) in mice, including **(A)** kidneys, **(B)** liver, **(C)** heart, **(D)** spleen, **(E)** lungs and **(F)** brain. Data are presented as mean ± SD (n = 4 biological replicates per group). Data analysis was performed by unpaired t-test with *p ≤ 0.05, **p ≤ 0.01, ***p ≤ 0.001.

**FIGURE 8:**
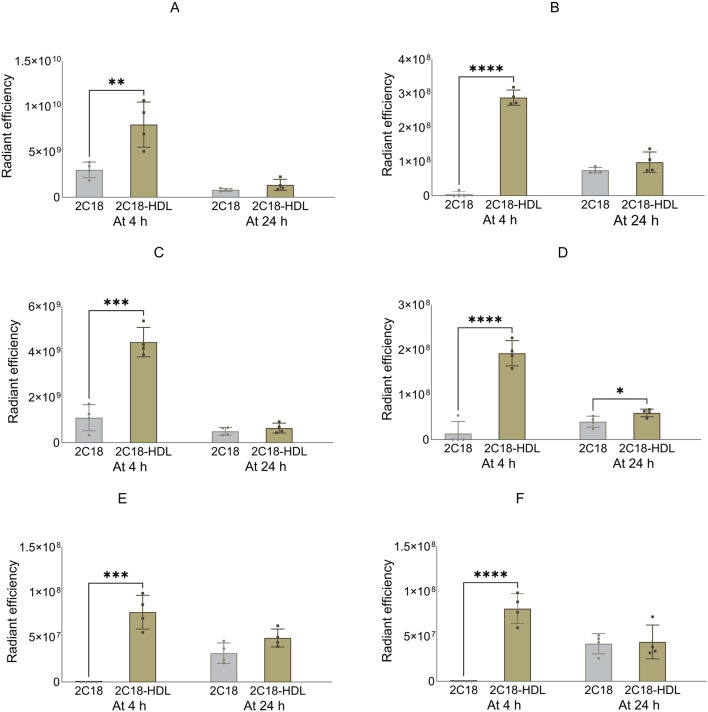
The radiant efficiency of harvested tissues at 4 or 24 h following SC dosing of 2C18-PEG (2C18) or 2C18-PEG/HDL (2C12/HDL) in mice, including **(A)** skin from the dosing site, **(B)** skin from the contralateral side, **(C)** muscle from the dosing site, **(D)** muscle from the contralateral side, **(E)** SC adipose tissue from the contralateral side and **(F)** mesenteric adipose tissue. Data are presented as mean ± SD (n = 4 biological replicates per group). Data analysis was performed by unpaired t-test with *p ≤ 0.05, **p ≤ 0.01, ***p ≤ 0.001, ****p ≤ 0.0001.

**FIGURE 9:**
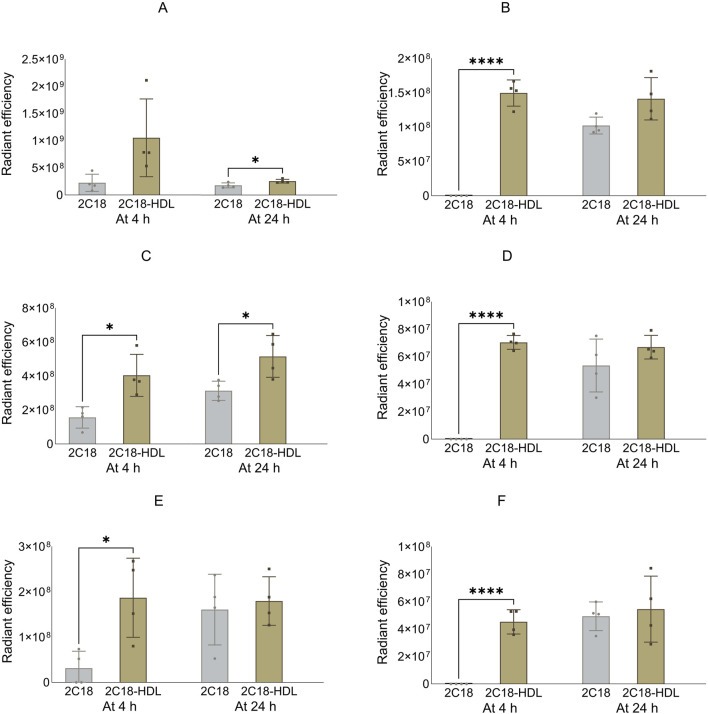
The radiant efficiency of harvested lymph nodes at 4 or 24 h following SC dosing of 2C18-PEG (2C18) or 2C18-PEG/HDL (2C12/HDL) in mice, including **(A)** popliteal lymph nodes from the ipsilateral side, **(B)** popliteal lymph nodes from the contralateral side, **(C)** inguinal lymph nodes from the ipsilateral side, **(D)** inguinal lymph nodes from the contralateral side, **(E)** iliac lymph nodes from the ipsilateral side and **(F)** iliac lymph nodes from the contralateral side. Data are presented as mean ± SD (n = 4 biological replicates per group). Data analysis was performed by unpaired t-test with *p ≤ 0.05, ****p ≤ 0.0001.

Next, the biodistribution of 2C18-PEG was compared in mice at 4 and 24 h after SC dosing with or without pre-mixing with HDL. Administration of 2C18-PEG with HDL resulted in several changes to the biodistribution of 2C18-PEG at 4 h and 24 h after SC dosing compared to 2C18-PEG in the absence of HDL (Figures 7–9). There was a significant increase in the apparent biodistribution of 2C18-PEG into the heart, spleen, lungs and brain at the 4 h timepoint on co-administration with HDL. At the 24 h timepoint, the apparent biodistribution of 2C18-PEG into the liver increased for 2C18-PEG/HDL compared with the 2C18-PEG.

The radiant efficiency of the skin and muscle from the ipsilateral side, skin and SC adipose tissue from the contralateral side, and mesenteric adipose tissue at the 4 h timepoint was higher in mice injected with 2C18-PEG/HDL compared with the corresponding tissues from mice injected with 2C18-PEG (Figure 8). In addition, 2C18-PEG had higher apparent distribution into the muscle from the contralateral side at 4 h and 24 h after dosing with the 2C18-PEG/HDL formulation compared with 2C18-PEG.

The apparent biodistribution of 2C18-PEG into all harvested lymph nodes (Figure 9), except the popliteal lymph nodes from the ipsilateral side, was enhanced at 4 h after dosing of 2C18-PEG/HDL compared with 2C18-PEG. In addition, 2C18-PEG/HDL enhanced the apparent biodistribution of 2C18-PEG into the ipsilateral popliteal and inguinal lymph nodes at 24 h after administration compared with 2C18-PEG. Altogether, pre-mixing with HDL appeared to increase the lymphatic uptake of 2C18-PEG which was the opposite of what was seen upon pre-mixing of 2C12-PEG with RSA.

Supplementary Figure S3 shows the radiant efficiency of the adipose tissue from the injection site at 24 h after SC dosing of 2C18-PEG and 2C18-PEG/HDL. There appeared to be no significant difference in the retention of 2C18-PEG in the adipose tissue at the injection site when administered with or without HDL. Despite this, administration of 2C18-PEG/HDL led to a higher plasma concentration of 2C18-PEG at the 24 h timepoint (but not 4 h) compared with conventional 2C18-PEG (Table 3) suggesting that the elimination phase was prolonged on dosing with HDL.

**TABLE 3:** Plasma concentrations of 2C18-PEG at 4 and 24 h after SC administration of 2C18-PEG and 2C18-PEG/HDL.

Plasma concentration (ng/mL)	2C18-PEG	2C18-PEG/HDL
4 h timepoint	4.2 ± 1.8	4.6 ± 0.5
24 h timepoint	2.9 ± 0.3^a^	5.3 ± 0.5^a^

^a^ significantly different compared with the other group (p ≤ 0.05).

Data are presented as mean ± SD (n = 4 biological replicates per group). Data analysis was performed by unpaired t-test.

### 3.5 SRB1 inhibition with BLT-1 alters the biodistribution of 2C18-PEG

**FIGURE 10:**
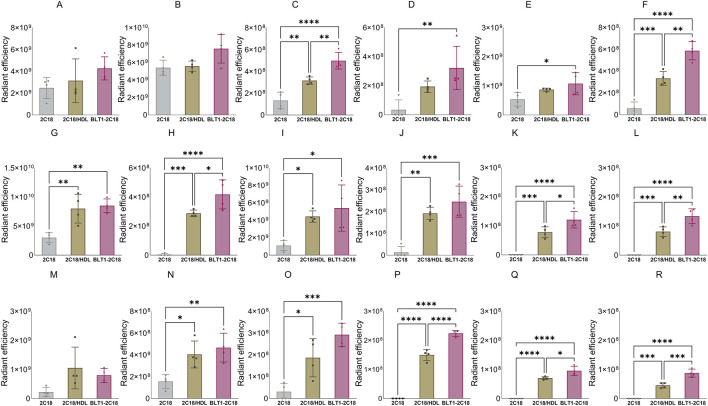
The biodistribution of 2C18-PEG into major organs (panels **(A–F)** in the first row), several tissues (panels **(G–L)** in the second row), and lymph nodes (panels M–R in the third row) at 4 h after SC dosing mice with conventional 2C18-PEG (2C18), 2C18-PEG/HDL (2C18/HDL) and BLT1-2C18-PEG (BLT1-2C18). Data are presented as mean ± SD (n = 4 biological replicates per group). Data analysis was performed by one-way ANOVA followed by Tukey’s multiple comparisons test with *p ≤ 0.05, **p ≤ 0.01, ***p ≤ 0.001, ****p ≤ 0.0001.

Figure 10 shows the biodistribution data for 2C18-PEG at 4 h after SC dosing in DPBS, pre-mixed with HDL (2C18-PEG/HDL) or 30 min following oral gavage of the SR-BI inhibitor BLT-1 (BLT1-2C18-PEG). First, the plasma concentrations of 2C18-PEG were not statistically different among the three groups at 4 h after dosing such that any difference in tissue biodistribution may not be well explained by differences in plasma exposure (Table 4).

**TABLE 4:** Plasma concentration of 2C18-PEG at 4 h after SC dosing mice with 2C18-PEG only, 2C18-PEG/HDL, and 2C18-PEG after pre-dosing with oral BLT-1 (BLT1-2C18-PEG).

	2C18-PEG alone	2C18-PEG/HDL	BLT1-2C18-PEG
Plasma concentration (μg/mL)	4.2 ± 1.8	4.6 ± 0.5	6.2 ± 2.5

Data are presented as mean ± SD, for groups (n = 4 biological replicates per group). Data analysis was performed by one-way ANOVA., there was no significant differences between the three groups.

The apparent biodistribution of 2C18-PEG into the heart, spleen, lungs and brain was higher when the rats were pre-dosed with BLT-1. Interestingly, the apparent biodistribution of 2C18-PEG into the heart and brain was also higher in the BLT1-2C18-PEG compared to the 2C18-PEG/HDL group. The skin and muscle tissues from the ipsilateral and contralateral sides had higher radiant efficiency in the BLT1-2C18-PEG group compared with matching tissues harvested from the mice that received 2C18-PEG alone. Moreover, the BLT1-2C18-PEG group had higher apparent biodistribution into the mesenteric adipose tissue on the contralateral side compared with the group that were dosed with 2C18-PEG alone and, intriguingly, the mice that were dosed with 2C18-PEG/HDL.

In the lymph nodes, the radiant efficiency of ipsilateral and contralateral inguinal and iliac lymph nodes was higher in the groups administered BLT1-2C18-PEG and 2C18-PEG/HDL compared to free 2C18-PEG. Also, the biodistribution of 2C18-PEG into all contralateral lymph nodes was higher in the BLT1-2C18-PEG group than the 2C18-PEG/HDL group. This data was somewhat unexpected as BLT1 was expected to inhibit the entry of 2C18-PEG into the lymphatics in association with HDL. However, BLT-1 might modulate the concentration and content of not only HDL but also other lipoproteins in biological fluids. So, triglyceride and cholesterol concentrations in plasma were measured in the three groups of mice (Supplementary Table S2). The plasma concentration of triglyceride (but not cholesterol) was statistically higher in the BLT1-2C18-PEG compared to the 2C18-PEG/HDL group suggesting that the differences in biodistribution in the BLT1 dosed group are a result of perturbations in lipoprotein trafficking.

## 4 Discussion

Identification that lymphatic dysfunction contributes to a wide range of diseases has led to a quest for new systems to deliver therapeutic, vaccine and imaging agents into the lymphatics. A new lymphatic delivery strategy that has materialized in recent years is to conjugate these agents or their delivery systems to ligands such as lipids that facilitate *in situ* association with endogenous albumin or lipoprotein trafficking pathways into lymphatics from tissues. Recently, we described a novel lymphatic delivery system in which brush PEG polymers are conjugated to different lipids (Abdallah et al., 2024a; Abdallah et al., 2024b). In our first studies, we demonstrated that the lipid conjugated brush PEG polymers can be recovered in association with albumin and lipoproteins in lymph fluid and plasma suggesting that they traffic into lymph in association with endogenous albumin and/or lipoproteins (Abdallah et al., 2024a; Abdallah et al., 2024b). However, it was not completely certain whether the polymers enter lymph predominantly in association with albumin and/or lipoproteins. The mechanism of lymphatic entry of the polymers is explored further in this paper as it may inform improved design of the brush PEG polymers and similar delivery systems. The current work also determines the impact of pre-mixing the lipidated polymers with exogenous albumin or lipoproteins on the polymer’s plasma pharmacokinetics, lymph uptake and/or biodistribution after SC and IV administration which could represent an alternate mode of delivery.

After administration in association with RSA, the IV elimination half-life of 2C12-PEG was significantly increased from 10.9 to 14.2 h, while the SC elimination half-life was extended from 17.0 h to 36.5 h. The extension of half-life may be related to protection against renal excretion due to the increase in complex size on association with RSA. We noted that the polymers are significantly excreted in urine with the Cy5 moiety turning the urine blue (Abdallah et al., 2024a; Abdallah et al., 2024b). Frich et al. also compared the SC plasma pharmacokinetic profiles and blood residence time of a poly(*N*-(2-hydroxypropyl) (PHPMA) polymer administered in unmodified form, conjugated with 1,2-distearoyl-sn-glycero-3-phosphoethanolamine (DSPE) lipid (DSPE-PHPMA), or conjugated to albumin (PHPMA-albumin) (Frich et al., 2019). Both lipidation and conjugation to albumin extended the plasma residence time compared to the unmodified PHPMA polymer, but there was no significant difference in the plasma residence time and pharmacokinetic profile between DSPE-PHPMA and PHPMA-albumin. The reason that pre-mixing of 2C12-PEG with RSA extended the plasma elimination half-life, differing to the study of Frich et al., could be related to the kinetics of association and dissociation with albumin where 2C12-PEG administered in the free form may take some time to bind to albumin leading to some pharmacokinetic and biodistribution behavior similar to unbound non-lipidated polymer. Alternatively, our recent study showed that a significant portion of 2C12-PEG is associated with lipoproteins such as HDL in lymph and plasma both *in vivo* and *in vitro*. This association with lipoproteins is also likely to impact the pharmacokinetics and biodistribution. Additionally, the concentration of RSA in our SC and IV formulations were 50 mg/mL and 15 mg/mL, which are higher and lower, respectively, than the concentration of albumin in the interstitium (16 mg/mL) and plasma (28 mg/mL) (Rutili and Arfors, 1977; Barber et al., 1990). The higher-than-endogenous level of albumin in the SC formulation of 2C12-PEG/RSA may have had an impact on the SC absorption profile and pharmacokinetics of 2C12-PEG.

Unexpectedly, the SC lymph uptake of 2C12-PEG was significantly reduced upon administration of 2C12-PEG/RSA compared to free 2C12-PEG. One potential explanation for this is that albumin is absorbed into blood capillaries after SC dosing. However, several studies have found that albumin is transported from the interstitium via lymphatics. For example, Triacca et al. demonstrated that albumin is transported from the interstitium into the lymphatic capillaries via both transcellular and paracellular pathways (Triacca et al., 2017). Wasserman and Mayerson found that radiolabeled albumin appeared in thoracic lymph within ∼10 min and for up to 5 days after IV administration (Wasserman and Mayerson, 1951). Furthermore, Patterson et al. found that 97% of a dose of albumin that was infused into the leg lymphatic vessels returned to the systemic circulation via the thoracic duct (Patterson et al., 1958). Taken together these studies support that albumin extravasates from blood capillaries into the interstitium and then distributes from the interstitium into lymphatic vessels before returning to blood again via the thoracic duct. Nevertheless, direct transport of albumin from the interstitium to blood capillaries cannot be ruled out. Leeds et al. evaluated the transport of radiolabeled albumin into lymph (collected via cannulation of the right lymphatic duct and thoracic duct) and blood after intrapericardial injection in dogs (Leeds et al., 1977). They found that radiolabeled albumin appeared in blood of lymph cannulated dogs, which indicated either that albumin is directly absorbed from the interstitium to blood capillaries, or indirectly transported from lymph to blood via lymphovenous communications, such as in lymph nodes (Hidden et al., 1985), prior to convergence of the lymphatics at the right lymphatic and thoracic duct (Leeds et al., 1977).

Consistent with the decrease in lymphatic uptake of 2C12-PEG when pre-mixed with RSA, the biodistribution to several tissues was significantly altered in mice, including a decrease in apparent distribution into popliteal lymph nodes, inguinal lymph nodes and skeletal muscles on the dosing side at 24 h after SC dosing of 2C12-PEG when pre-mixed with RSA. In contrast, Frich et al. found that PHPMA-albumin had higher apparent accumulation in lymph nodes, liver, spleen and lungs compared with DSPE-PHPMA at 24 h and 7 days after SC dosing (Frich et al., 2019). Importantly, this study did not mention the name or site of the analyzed lymph nodes, and also, they did not clearly specify whether the harvested lymph nodes were injection-site draining lymph nodes, non-draining lymph nodes or both. Overall, our findings provide new evidence about the mechanism of entry of the polymers into the lymphatics as it appears that hitchhiking on albumin cannot fully explain the lymph uptake of the polymers because pre-mixing with albumin reduces, not increases, lymphatic uptake.

In our previous study, we showed that a significant proportion of the lipidated brush PEG polymers was recovered in both the lymph fluid and plasma in association with HDL, suggesting that direct uptake into lymph may occur in association with HDL. Next, we therefore examined the impact of pre-mixing 2C18-PEG with HDL (2C18-PEG/HDL) on the biodistribution, plasma exposure and lymph node uptake of the polymer. Pre-mixing with HDL was associated with many changes to the biodistribution of 2C18-PEG following SC injection, including an increase in concentrations in the dosing-side ipsilateral inguinal, iliac and/or popliteal lymph node at the 4 and/or 24 h timepoint. Further, concentrations of 2C18-PEG pre-mixed with HDL were higher in the dosing-side ipsilateral compared to the contralateral inguinal, iliac and/or popliteal lymph nodes after SC injection supporting that 2C18-PEG/HDL directly enters the lymphatics. The plasma concentration of 2C18-PEG was also higher at 24 h when dosed with HDL suggesting that the pharmacokinetics may be altered with the elimination half-life extended for 2C18-PEG/HDL compared to 2C18-PEG. While the concentration of HDL (10 mg/mL) in the formulation was higher than that expected in tissues or plasma (∼1 mg/mL in mice) (Bérard et al., 1997), these studies do suggest that pre-mixing with HDL could be used to boost lymphatic uptake and extend the pharmacokinetic profile of lipid conjugated polymers or similar delivery systems. Further, the findings support that lymphatic uptake of the lipid conjugated polymers is higher when they hitchhike on HDL rather than albumin trafficking pathways.

The different effects of pre-mixing 2C12-PEG with RSA and 2C18-PEG with HDL on lymph uptake is intriguing. This unexpected finding suggests that albumin and HDL might have different absorption pathways from the interstitium. As Leeds et al. demonstrated, a direct absorption of albumin from the injection site in the interstitium into the blood circulation is possible, although lymph absorption is still the main pathway of albumin absorption (Leeds et al., 1977). On the other hand, there is no evidence in the literature that HDL can be absorbed directly into the blood circulation from the interstitium. The size of L-HDL (spherical particles with a diameter of 8.9–17.8 nm) is relatively similar to albumin (ellipsoid shape particles with an approximate size of 3.8 × 15 nm) (Abdallah et al., 2020; Gracia et al., 2020). Thus, size is not likely the driving force that controls different absorption pathways of L-HDL and albumin. Known active transport pathways for albumin such as neonatal Fc receptor (FcRn) might facilitate the transport of albumin into blood from the interstitium (Abdallah et al., 2020); however, the involvement of these pathways in the transport of albumin from the interstitium across blood capillaries has not been confirmed yet.

To further explore whether the lymphatic uptake of the polymers is mediated by hitchhiking on lipoprotein trafficking pathways, a further study was performed where the SRB1 inhibitor BLT-1 was administered by oral gavage to mice prior to SC administration of 2C18-PEG and the impact on 2C18-PEG biodistribution was determined. SRB1 facilitates the entry of cholesterol esters from the core of HDL particles into cells (Shen et al., 2014). Importantly, a study conducted by Lim *et al* found that SRB1 facilitates the transcellular transport of HDL particles from the interstitium into the lymphatics and that BLT-1 can inhibit HDL entry into lymphatics (Lim et al., 2013). Surprisingly, pre-dosing BLT-1 led to higher rather than lower uptake of 2C18-PEG into the inguinal and iliac lymph nodes from both the dosing and non-dosing side with higher concentrations observed on the dosing-side suggesting direct lymph uptake. Additionally, mesenteric and SC adipose tissue from the contralateral side of mice that received BLT-1 prior to dosing with 2C18-PEG demonstrated higher radiant efficiency than the same tissues collected from mice dosed with 2C18-PEG alone and 2C18-PEG/HDL. Whilst we expected to observe a reduction rather than increase in lymphatic uptake of 2C18-PEG after pre-dosing BLT-1, this result shows that perturbing lipoprotein and lipid trafficking profiles impacts the lymphatic uptake of 2C18-PEG. In addition to inhibiting HDL entry into the lymphatics, SRB1 also binds with LDL particles and may change the concentration and/or biodistribution of LDL particles (Yang et al., 2013). BLT-1 has poor water solubility such that we orally administered BLT-1 in a vehicle composed mostly of corn oil (90% v/v), which is rich in long-chain fatty acids that can increase intestinal chylomicron formation and consequently CM concentrations in mesenteric lymph and plasma (Barrera-Arellano et al., 2019). Indeed, plasma samples from mice pre-dosed with BLT-1 had higher triglyceride concentrations than mice that received 2C18-PEG/HDL but not the mice that received 2C18-PEG alone. Overall, the oral pre-dose with BLT-1 would have perturbed the concentration, composition and trafficking of several lipoprotein classes in lymph and plasma. Previously we demonstrated that 2C18-PEG can be found in lymph and plasma in association with HDL, LDL and VLDL/chylomicrons as well as a small fraction on albumin (<20%). BLT-1 thus likely altered the lymphatic uptake of 2C18-PEG by altering its trafficking into lymph in association with different lipoprotein particles.

The binding of pharmaceutical agents with endogenous proteins such as albumin has been widely employed to overcome many challenges in drug delivery (Larsen et al., 2016). As an example, binding with albumin has been translated clinically into parenteral formulations to extend the elimination half-life of pharmaceutical agents, and also to deliver pharmaceutical agents to the lymphatic system for lymph-related diagnostic purposes (Larsen et al., 2016; Abdallah et al., 2020). Yet, employing endogenous proteins to achieve improvements in drug delivery may be limited. The level of endogenous serum albumin in the body can be reduced in many illnesses such as malnutrition, liver diseases, cancers, trauma and nephrotic syndrome due to decreased synthesis, excessive excretion and/or enhanced vascular permeability (Van Tongeren et al., 1978; Soeters et al., 2019). This could affect the pharmacokinetic profiles of therapeutics that bind extensively with endogenous albumin, leading to altered pharmacological properties such as the elimination half-life (Tayyab and Feroz, 2021). Advanced drug delivery systems incorporating exogenous albumin might have the potential to ameliorate the drawbacks of hypoalbuminaemia and drug-drug interactions on the pharmacokinetics of pharmaceutical agents that bind to endogenous albumin (Vishnu and Roy, 2011; Larsen et al., 2016; Hassanin and Elzoghby, 2020; Spada et al., 2021).

Exploiting exogenous albumin in drug delivery is not entirely free from complications and issues. The use of bovine serum albumin (BSA) in humans might provoke immune responses (Hassanin and Elzoghby, 2020). Also, the synthesis of albumin-based nanoformulations can be expensive and could entail toxic organic reagents such as glutaraldehyde, or might involve steps (e.g., high-pressure homogenization) that could affect the stability of albumin (Hassanin and Elzoghby, 2020). Similar to exogenous albumin, exogenous lipoproteins might be associated with complications such as altered lipid metabolism (Busatto et al., 2020). More studies are warranted to evaluate the long-term efficacy and safety of exogenous lipoprotein-based drug delivery systems.

It should be pointed out that our present study has limitations. As mentioned in section 2.3, the unbound fraction of 2C12-PEG and 2C18-PEG after mixing with RSA and HDL, respectively, could not be filtered. Unfortunately, the polymers became adsorbed onto different filter membranes (made of regenerated cellulose or polyethersulfone) when we utilized ultrafiltration methods to filter the unbound fraction of the polymers, and also to measure the unbound vs. bound fraction. Nonetheless, the free fraction of 2C12-PEG and 2C18-PEG after mixing with albumin and lipoproteins is likely to be very low. This has been demonstrated in our previously published papers using an ultracentrifugation-based assay, in which there was a remarkable change in the association pattern of the polymers with different protein and lipoprotein fractions of rat lymph and plasma from fed and fasted rats when compared with the association pattern of the polymers with RSA solution alone (Abdallah et al., 2024a; Abdallah et al., 2024b). In future studies, alternative methods such as size-exclusion chromatography, capillary electrophoresis, and high-performance affinity chromatography should be employed to accurately quantity the unbound fraction of the polymers, and also to determine other binding parameters such as the number of binding sites and the association and dissociation rate constants. Another limitation is the absence of a conclusive investigation that confirms the alteration in the profiles of lipoproteins and the association of 2C18-PEG with different lipoproteins in the group of mice dosed orally with the SRB1 inhibitor BLT-1. This investigation could be carried out in the future to provide additional evidence for the suggested mechanism of the enhanced lymph transport of 2C18-PEG in mice dosed with BLT-1.

## 5 Conclusion

Pre-mixing of the diacylglycerol-conjugated brush polymer delivery systems (2C12-PEG and 2C18-PEG) with exogenous albumin and HDL prolonged its elimination half-life and plasma exposure which may have benefits in reducing dosing frequency for patients. Interestingly, pre-mixing 2C12-PEG with RSA was associated with lower lymph uptake following SC dosing compared with free 2C12-PEG. By contrast, pre-mixing 2C18-PEG with HDL particles altered its tissue biodistribution profile, including an increase in delivery to lymph nodes. Pre-mixing with HDL could thus be exploited to increase lymphatic delivery. Finally, pre-administration of the SRB1 inhibitor BLT-1 increased distribution of 2C18-PEG into dosing-side inguinal and iliac lymph nodes and adipose tissues which is presumably a result of perturbations to the concentration, composition and/or trafficking of HDL, LDL, VLDL and/or CM in lymph and plasma. Altogether these studies show that the trafficking pathways of the diacylglycerol-conjugated brush polymer delivery systems is far more complex than initially anticipated and certainly does not involve only hitchhiking on albumin trafficking pathways into lymph. Importantly, these studies suggest that manipulating the properties of the delivery system to increase association with HDL trafficking pathways could be a viable means to enhance lymphatic uptake further and provide more specific and effective diagnostic or treatment agents for lymphatic related diseases.

## Data Availability

The original contributions presented in the study are included in the article/Supplementary Material, further inquiries can be directed to the corresponding authors.
