# Supplementary material for: Lymphatic uptake and pharmacokinetics of lipid conjugated brush PEG polymers is altered by interactions with albumin and lipoproteins
Source: Front Physiol. 2025 Jun 27;16:1610791. doi: 10.3389/fphys.2025.1610791 (PMC12245853; doi:10.3389/fphys.2025.1610791)
Supplement: Supplementary file 1 [file DataSheet1.docx]

Supplementary Material

# Supplementary Figures and Tables

## Supplementary Figures

**Supplementary Figure 1.** The apparent elimination (terminal) half-life of the two 2C12-PEG formulations (2C12-PEG and 2C12-PEG/RSA) following IV and SC dosing to rats. Data are presented as mean ± SD for groups (n=3 biological replicates). Statistical analysis was performed by two-way ANOVA followed by Tukey’s multiple comparisons test to compare between the IV and SC elimination half-life of each formulation. The difference in the elimination half-life between the two formulations is not shown in this figure. **P ≤ 0.01, ****P ≤ 0.0001.

**Supplementary Figure 2.** The radiant efficiency of adipose tissue harvested from the SC injection site at 24 h after dosing the two 2C12-PEG formulations (2C12-PEG and 2C12-PEG/RSA). There was no significant difference in the apparent retention of 2C12-PEG in the SC adipose tissue from the injection site between the two formulations. Data are presented as mean ± SD for groups (n=4 biological replicates). Data analysis was performed by unpaired t-test.

**Supplementary Figure 3.** The radiant efficiency of adipose tissue harvested from the SC injection site at 24 h after dosing the two 2C12-PEG formulations (2C18-PEG and 2C18-PEG/HDL) SC. There was no significant difference in the apparent retention of 2C18-PEG in the SC adipose tissue from the injection site between the two formulations. Data are presented as mean ± SD for groups (n=4 biological replicates). Data analysis was performed by unpaired t-test.

## Supplementary Tables

| **Supplementary Table 1.** Equations used to calculate non-compartmental plasma pharmacokinetic parameters after IV and SC administration of 2C12-PEG formulations | |
| --- | --- |
| **Pharmacokinetic parameter** | **Method or equation** |
| Initial plasma concentration (Cp^0^) (for IV pharmacokinetic studies) | Estimated by a regression of the 1 and 5 mins timepoints |
| AUC_0 –_ _last timepoint_ | Determined by the linear trapezoidal rule |
| AUC_last timepoint – infinity_ | (Last measurable plasma concentration)/b |
| AUC_0 – infinity_ or AUC_Total_ | AUC_0 –_ _last timepoint_ + AUC_0 – infinity_ |
| b (elimination rate constant) | Slope of the log_e_ plasma concentration vs time profile during the terminal elimination phase |
| Plasma half-life | 0.693/b |
| Total body clearance (IV pharmacokinetic studies) | Dose/AUC_Total_ |
| Vd during the terminal phase (IV pharmacokinetic studies) | Dose/(b x AUC_Total_) |
| Bioavailability (SC pharmacokinetic studies) | AUC_Total_ SC/AUC_Total_ IV |
| AUC: area under the plasma-concentration time curve, Vd: volume of distribution. | |

| **Supplementary Table 2.** Plasma concentration (mg/ml) of triglyceride and cholesterol at four h after dosing mice with 2C18-PEG only, 2C18-PEG-HDL and BLT1-2C18-PEG | | | |
| --- | --- | --- | --- |
| Lipid | Mice group | | |
|  | 2C18-PEG only | 2C18-PEG/HDL | BLT1-2C18-PEG |
| Triglyceride | 0.5 ± 0.07 | 0.2 ± 0.05^b^ | 1.1 ± 0.58^a^ |
| Cholesterol | 1.0 ± 0.34 | 0.8 ± 0.14 | 0.7 ± 0.24 |
| Data are presented as mean ± SD for groups (n=4 biological replicates). Data analysis was performed by one-way ANOVA followed by Tukey’s multiple comparisons test. ^a^significantly different compared with 2C18-PEG/HDL (P ≤ 0.05). ^b^significantly different compared with BLT1-2C18-PEG (P ≤ 0.05). | | | |
